# Digital measurement method for comparing the absolute marginal discrepancy of three-unit ceramic fixed dental prostheses fabricated using conventional and digital technologies

**DOI:** 10.1186/s12903-023-03620-9

**Published:** 2023-11-17

**Authors:** Shanshan Liang, Fusong Yuan, Deli Li, Lu Jia, Yuchun Sun

**Affiliations:** 1 Center of Digital Dentistry/Department of Prosthodontics/Second Clinical Division, Peking University School and Hospital of Stomatology & National Center of Stomatology & National Clinical Research Center for Oral Diseases &National Engineering Research Center of Oral Biomaterials and Digital Medical Devices & Beijing Key Laboratory of Digital Stomatology & Research Center of Engineering and Technology for Computerized Dentistry Ministry of Health, Beijing, 100081 People’s Republic of China; 2 Center of Digital Dentistry/Department of Prosthodontics, Peking University School and Hospital of Stomatology & National Center of Stomatology & National Clinical Research Center for Oral Diseases &National Engineering Research Center of Oral Biomaterials and Digital Medical Devices & Beijing Key Laboratory of Digital Stomatology & Research Center of Engineering and Technology for Computerized Dentistry Ministry of Health, Beijing, 100081 People’s Republic of China; 3 Second Clinical Division, Peking University School and Hospital of Stomatology & National Clinical Research Center for Oral Diseases & National Engineering Research Center of Oral Biomaterials and Digital Medical Devices & Beijing Key Laboratory of Digital Stomatology, Beijing, 100081 People’s Republic of China; 4 Denture Processing Center, Peking University School and Hospital of Stomatology, & National Clinical Research Center for Oral Diseases & National Engineering Research Center of Oral Biomaterials and Digital Medical Devices & Beijing Key Laboratory of Digital Stomatology, Beijing, 100081 People’s Republic of China

**Keywords:** Marginal fit, Absolute marginal discrepancy, Digital evaluation, Fixed dental prostheses

## Abstract

**Background:**

In clinical practice, control of the marginal fit of fixed dental prostheses is hindered by evaluation method, which needs to be further improved to increase its clinical applicability. This study aimed to quantitatively analyze the absolute marginal discrepancy of three-unit ceramic fixed dental prostheses fabricated by conventional and digital technologies using a digital measurement method based on the digital impression technology and open source software.

**Methods:**

A digital workflow and the conventional impression combined with the lost-wax heat-pressed technique were adopted to separately fabricate 10 glass ceramic fixed dental prostheses. Three-dimensional data for the abutments, fixed dental prostheses, and fixed dental prostheses seated on the abutments, were obtained using a dental scanner. The two datasets were aligned using registration technology, specifically “multi-points registration” and “best fit alignment,” by reverse engineering software. Subsequently, the three-dimensional seated fit between the fixed dental prostheses and abutments were reconstructed. The margin of the abutment and crown was extracted using edge-sharpening and other functional modules, and the absolute marginal discrepancy was measured by the distance between the margin of the abutment and crown. One-way analysis of variance was used to statistically analyze the measurement results.

**Results:**

Using the digital measurement method, the mean value of absolute marginal discrepancy for fixed dental prostheses fabricated by the conventional method was 106.69 ± 6.46 μm, and that fabricated by the digital workflow was 102.55 ± 6.96 μm. The difference in the absolute marginal discrepancy of three-unit all-ceramic fixed dental prostheses fabricated using the two methods was not statistically significant (*p* > 0.05).

**Conclusions:**

The digital measurement method for absolute marginal discrepancy was preliminarily established based on open source software and applied in three-unit ceramic fixed dental prostheses. The absolute marginal discrepancy of three-unit ceramic fixed dental prostheses fabricated using digital technology was comparable to that of conventional technique.

## Background

Three-unit fixed dental prostheses (FDPs) remain the primary restoration method for patients with the loss of a single tooth. However, the long-term outcomes of FDPs, particularly the stability, may be influenced by their marginal adaptation [1–3]. An excessive marginal discrepancy between the restoration and abutment may increase the risk of secondary caries, dental plaque accumulation, and periodontal diseases [1, 3].

Marginal fit is a key indicator of the accuracy of FDPs [1–4]. The criteria for evaluating the marginal fit include marginal discrepancy, vertical marginal discrepancy, and absolute marginal discrepancy (AMD) [5]. AMD is the distance between the margins of the retainer and the tooth preparation and is the maximum value of the marginal discrepancy measurement, reflecting the total misfit at a particular point; any other measure of fit may conceal the marginal discrepancies that actually exist [5]. A large AMD indicates that the margin of the retainer is placed too far from the margin of the tooth preparation, which can lead to plaque accumulation and periodontal disease. Therefore, the usability of such fixed restorations should be evaluated [6, 7].

Various methods have been proposed for the measurement of marginal discrepancy [8–13], each with their advantages and limitations; however, no unified standard exists [11–14]. Currently, the most common method of the quantitative analysis of marginal discrepancy is the replica technique [9, 10]. A silicone impression material is injected into the tissue surface of the restoration, which is then placed on the prepared tooth to record the gap between the prosthesis and the prepared tooth; this gap is then measured using a microscope. However, a disadvantage of this method is that bubbles or defects may exist at the site of interest in the silicone impression [15]. Furthermore, sectioning of the impression for in vitro measurements may cause deformation, preventing accurate measurements in some sites [16].

With the widespread application of digital technology in the dental field, it is also being used to measure the marginal fit of dental restorations [12, 13]. Liang et al. reported a fully digital method of marginal fit evaluation of a single crown that can provide more comprehensive measurement data [17].

Recently, fully digital workflows are being widely used for the fabrication of ceramic restorations [18, 19]. However, errors may be introduced in every step of the scanning, designing, and milling processes [20–22]. Currently, some studies has investigated the vertical marginal and the internal fit of FDPs [23, 24]. However, there is no relevant published literature on the use of digital measurement methods to analyze the AMD of three-unit ceramic FDPs. In clinical practice, the control of the marginal fit of FDPs is limited by the evaluation method. Therefore, further research on the digital measurement method for three or more unit FDPs is needed.

Thus, this study aimed to quantitatively analyze AMD of three-unit ceramic FDPs fabricated by conventional and digital technologies using a digital measurement method based on the digital impression technology and open source software. The null hypothesis was that there was no significant difference in the AMDs of three-unit ceramic FDPs fabricated using the two technologies .

## Methods

### Simulating the chairside environment

**Fig. 1:**
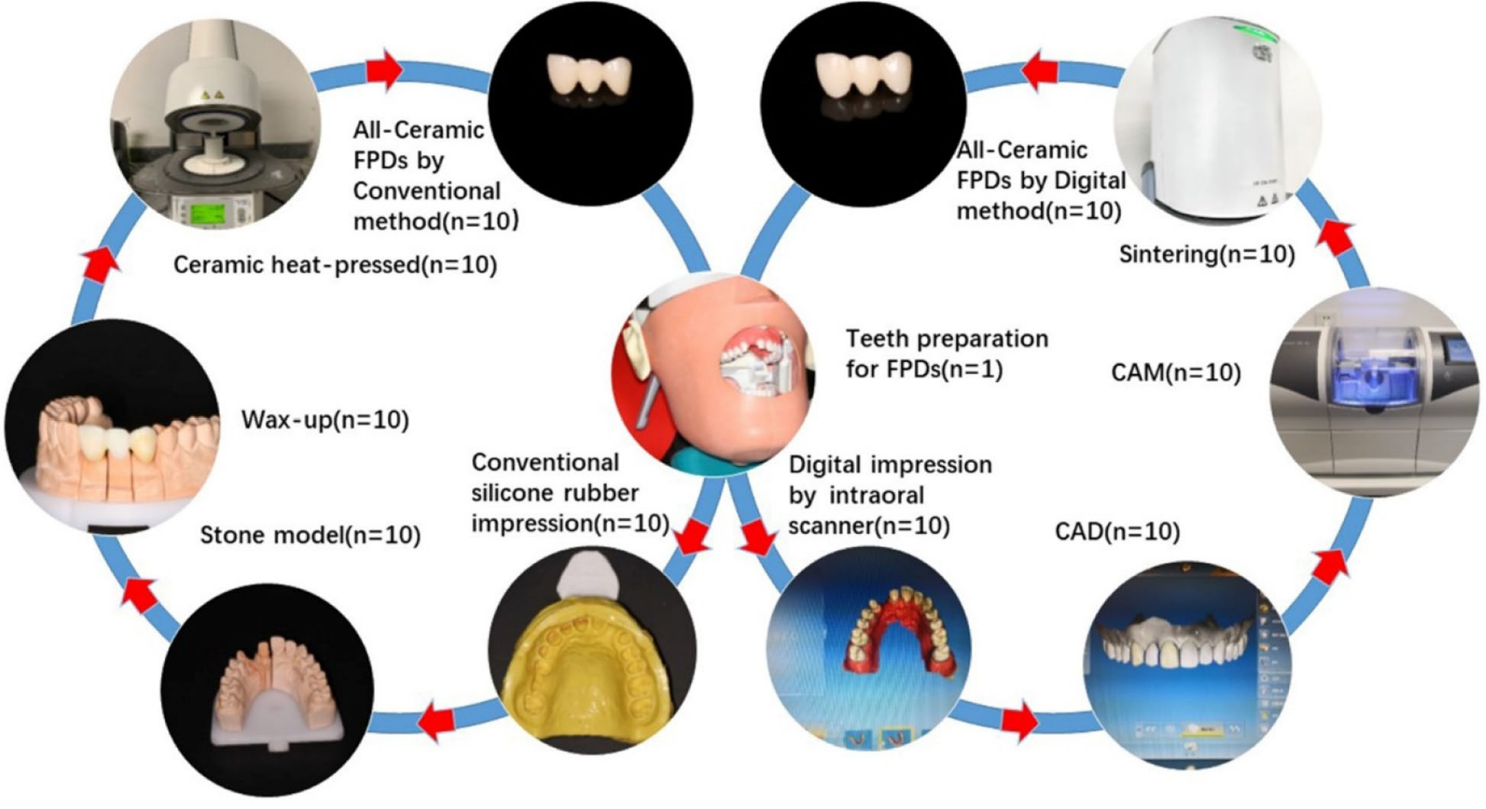
Conventional and chairside CAD/CAM fabrication processes for FDPs.  FDP, fixed dental prostheses; CAD/CAM, computer-aided design/computer-aided manufacturing

This study was approved by the Bioethics Committee of the Stomatological Hospital of Peking University (Beijing, China; No: PKUSSIRB-202059170; Date: November 18, 2020). A standard dentition model (A50 SET, Nissin, Tokyo, Japan) was used for this study. The maxillary right lateral incisor (FDI 12) was missing, and the maxillary right central incisor (FDI 11) and canine (FDI 13) were prepared to receive a three-unit all-ceramic FDP. An optical stereomicroscope (SZX7, Olympus, Tokyo, Japan) was used at 10× magnification to confirm that the margin of the tooth preparation was clearly visible and smooth, with a continuous 90° shoulder. The standard dentition model was placed in a mannequin head (Type 1 Advance, Nissin, Tokyo, Japan) mounted on a dental chair. The details of the fabrication process are shown in Fig. 1.

### Fabrication of FDPs using the conventional method

Ten polyether impressions (Impregum Penta Soft, 3 M ESPE, MN, USA) were obtained and stored at 35 ± 1 °C for 15 min to simulate the intraoral temperature [23, 24]. After storage at room temperature for 8 h, the 10 impressions were poured using type IV gypsum (Die Stone Peach, Kulzer GmbH, Hanau, Germany) [25], mixed according to the manufacturer’s instructions, to obtain 10 models. The gypsum models were separated from the impression after 40 min and stored at 25 ± 1 °C for 48 h. Die spacers (gold, YETI, dentalprodukte GmbH, Germany) were coated on the abutment teeth on the models, and 10 wax patterns were prepared by hand for fabricating glass ceramic FDPs (E.max press, Ivoclar Vivadent AG, Liechtenstein) using the conventional lost-wax heat-pressed technique.

### Fabrication of FDPs using the digital workflow

A chairside intraoral scanner (CEREC Omnicam, Dentsply Sirona, Germany) was used to scan the abutment teeth in the model in the mannequin head, and a computer-aided designing (CAD) software (CEREC 4.5.2, Dentsply, Sirona Germany) was used to design a three-unit all-ceramic FDP. The thickness of the virtual cement layer was set to 50 μm, and the cross-sectional area of the connector body was set to at least 9 mm^2^. Ten three-unit all-ceramic FDPs were fabricated using the CEREC chairside system with lithium disilicate-reinforced glass ceramics.

The scan process was performed under uniform conditions of ambient light by the same skilled dentist with more than 10 years of experience to control the accuracy of scanning. The FDPs were fabricated by the same dental technician with 15 years of experience to ensure quality.

### Digital measurement method for marginal fit of FDPs

**Fig. 2:**
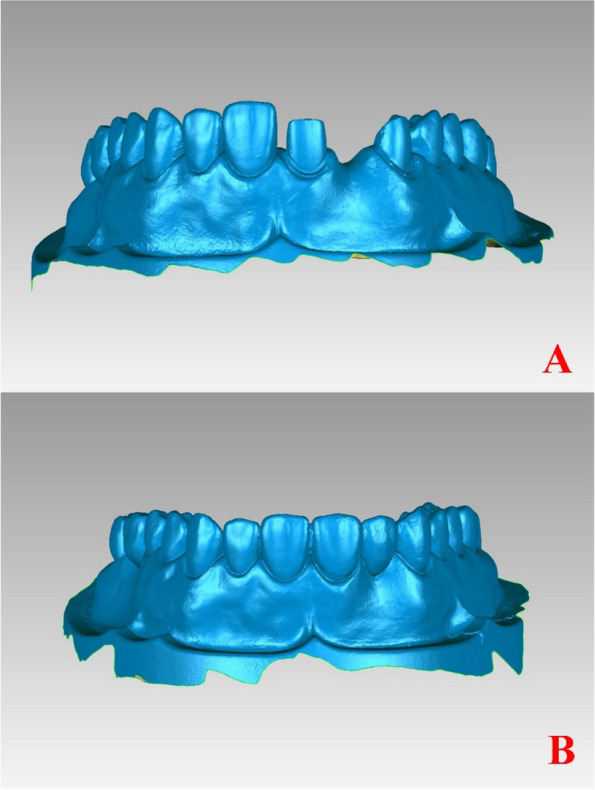
Two scans of the dentition at the site of planned fixed dental prostheses in the same coordinate system. A. After preparation of abutment teeth; B. After the fixed partial denture is seated

When the chairside intraoral scanner is used according to the manufacturer’s instructions, the scanning accuracy is 20 μm. First, the standard model with the abutment teeth was rescanned to ensure consistency of evaluation. Second, the intaglio surface of the FDP was scanned before it was seated on the abutments, and then, the external surfaces of the retainers and the pontic were scanned. Finally, a high-fluidity light-bodied vinyl polysiloxane impression material (Type 3, Imprint II Garant, 3 M ESPE, MN, USA) was injected into the intaglio surface of the FDPs, the FDPs was placed on the abutment teeth. Then a 500 g weight was kept on the occlusal surface for 5 min until the vinyl polysiloxane impression was completely polymerized, and the excess impression material (Type 3, Imprint II Garant, 3 M ESPE, MN, USA) on the margins of the FDP was removed. The external surface of the FDP was scanned. Clear scanning results were obtained without the need for powder coating. All of the above three datasets were stored in the standard tessellation language (STL) format for 3D point cloud data (Fig. 2a, b).

**Fig. 3:**
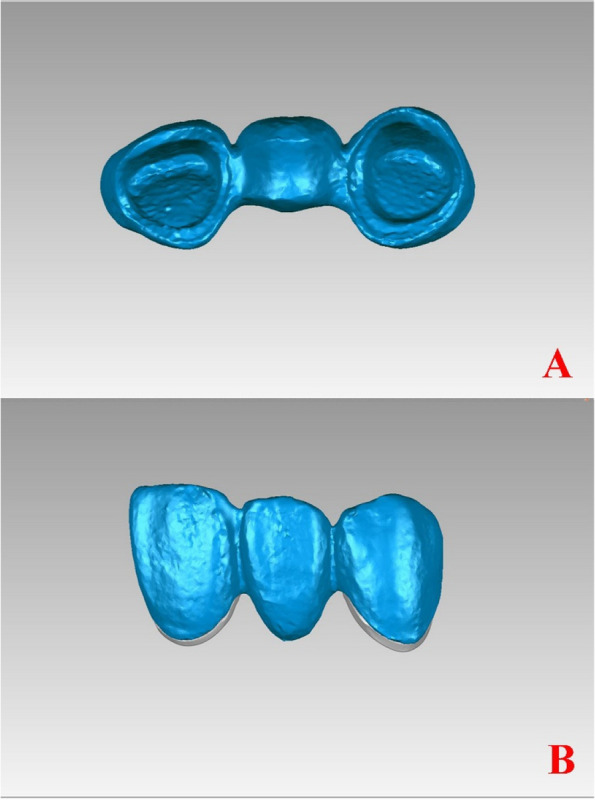
Virtual positioning of the fixed dental prostheses through registration in the same coordinate system. A. Before virtual positioning of the fixed partial denture; B. After virtual positioning of the fixed partial denture

A reverse engineering software (Geomagic Studio 2013, 3D Systems, Rock Hill, SC, USA) was used to analyze the above-mentioned datasets. Through two pairwise data alignments, the virtual 3D seating fit between abutments and the FDP was measured (Fig. 3a, b). These two datasets were aligned a combination of “multi-points registration” and “Best Fit Alignment.” The initial position was selected interactively, and two STL data files were preliminary aligned in the same coordinate system using the “muti-points registration” module, and the alignment was optimized using the “Best Fit Alignment” module, which is based on the iterative closest point algorithm.

**Fig. 4:**
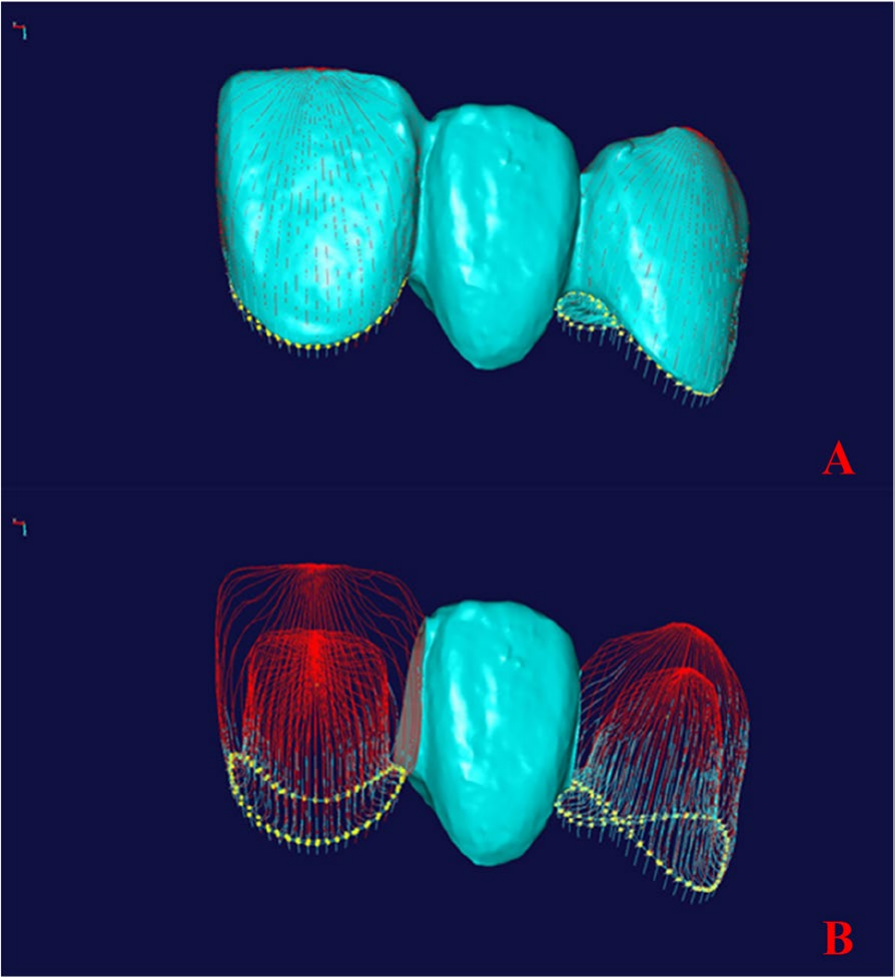
The digital measurement method for absolute marginal discrepancy. **A** Determination of the central axis, circular sectioning, and edge extraction; **B** Measurement of absolute marginal discrepancy

The edge-sharpening module of the open source software (Imageware v13.0, Siemens, Germany) was used to extract the edges of the prepared abutments and retainers of the FDP. Interactive selection was used to confirm the long axes of registered prepared abutments and retainers. These long axes were considered the central axis of each retainer and its corresponding abutment of the FDP. The abutments and their corresponding FDP retainers were equally circumscribed into 25 sections along the central axis, and 50 curves were obtained for each retainer and the corresponding abutment. The outer edges of the retainer and abutment shoulders were extracted and intersected with the 50 curves to obtain 100 points. The straight-line distance between the outer edge points on each FDP retainer and the corresponding points on the abutment, namely the AMD, was measured (Fig. 4a, b). All measurements were performed by the same observer with more than 10 years of experience using reverse engineering software (Geomagic Studio 2013, 3D Systems, Rock Hill, SC, USA).

### Statistical analysis

Normality and variance of the AMD data were evaluated with the Shapiro-Wilk and Levene tests using the SPSS version 26 (IBM, Armonk, NY, USA). Mean and standard deviation of the obtained values were calculated, and one-way analysis of variance (ANOVA) was used to evaluate the differences in the AMD between digital and conventional methods of three-unit all-ceramic FDPs. The sample size (*n* = 10) was based on our pilot study. A priority power analysis was performed by PASS 15.0 software program (Power Analysis and Sample Size 15.0, NCSS Statistical Software, Utah, USA) to ensure statistical significance (α = 0.05) at 80% power. The significance level was set at *p* < 0.05, with a 95% confidence interval.

## Results

The AMDs of the FDPs are presented in Tables 1 and 2. The AMD of three-unit all-ceramic FDPs fabricated using the conventional method was 106.69 ± 6.46 μm, whereas that of three-unit all-ceramic FDPs fabricated using the digital workflow was 102.55 ± 6.96 μm. The marginal discrepancy of the FDPs fabricated using both methods was within the clinically acceptable range. One-way ANOVA showed that the AMDs of FDPs fabricated using conventional and digital technologies were not statistically different (*p > 0.05*).

**Table 1:** Descriptive statistics of the absolute marginal discrepancy (μm) of three-unit all-ceramic fixed dental prostheses fabricated using CM and DM

	N	Mean	Standard Deviation	Standard Error	95% Confidence Interval of the Mean		Minimum	Maximum
					Lower Bound	Upper Bound		
CM	10	106.69	6.46	2.04	102.07	111.31	96.00	114.00
DM	10	102.55	6.96	2.20	97.5	107.53	91.40	111.90
Total	20	104.62	6.87	1.54	101.40	107.84	91.40	114.00

CM, conventional method; DM, digital method

**Table 2:** One-way analysis of variance of the mean absolute marginal discrepancy (μm) of three-unit all-ceramic fixed dental prostheses fabricated using conventional and digital methods

	Sum of Squares	df	Mean Square	F	*p*-value
Between groups	85.94	1	85.94	1.91	.18
Within groups	811.56	18	45.09		
Total	897.50	19			

df, degrees of freedom

## Discussion

A digital method for the quantitative analysis of three-unit FDPs fabricated using digital and conventional methods was developed. The results showed no statistically significant difference between the AMDs of FDPs fabricated using the digital and conventional methods. Thus, the null hypothesis was accepted.

FDPs must have a clinically acceptable marginal fit on all abutments. Dahl et al. emphasized that the suitability of three-unit FDPs for clinical use depends on the coordination between the two retainers. In three-unit FDPs, the misfit of any one retainer may hinder the complete seating of the other [26]. In the present study, both retainers of each FDP had the same path of insertion. Irrespective of whether a digital or conventional fabrication method was used, the AMD values for both retainers were less than 120 μm, which is within the clinically acceptable range [8, 25].

Papadiochou et al. found that most heat-pressed lithium disilicate crowns have a marginal fit equivalent to or better than that of CAD/CAM crowns [3]. Lo Russo et al. analyzed data on vertical marginal fit and found no statistically significant difference in the vertical marginal fit of FDPs fabricated using digital and conventional workflows [24]. Some studies reported that fully digital workflows yielded restorations with comparable or better marginal discrepancy than heat-pressed methods [27–29]. In this study, one-way ANOVA indicated no statistical difference between the conventional and digital methods. The result is consistent with the above literature [24, 27, 29].

The conventional method of FDP fabrication includes several primary steps, such as making silicone impressions [30], pouring gypsum molds [31], preparing wax patterns [32], and casting using the lost-wax heat-pressed technique [33]. Errors can be introduced in any step, which can affect the extent of cement space and fit of the final restoration [30–33].

In the digital workflow, the cement space can be set accurately. The type and accuracy of the scanner, use of powder coating, selection of restorative materials, and accuracy of 3D data alignment affect the marginal fit of the restoration [4, 27, 34–36].

Currently, no uniform standards exist for the measurement of marginal discrepancy of restorations, and the most common method is the replica technique [8, 9]. However, difficulties exist in the actual application of the replica technique. In most studies, only two to four measurement points were used to assess marginal discrepancy [25], which means that some sites were not evaluated [17].

Regardless of the method used to measure marginal discrepancy, selection of correct measurement points is essential. In the present study, 50 measurement points equally divided between different areas were selected based on the points used by Groten et al. [37]. The main limitations of silicone impression and replica techniques have been effectively overcome through the use of a fully digital method, which can achieve consistent and precise selection of measurement points. The number of available measurement points was unlimited,of which 50 points were selected evenly on a 360° arc, and the selection was not affected by occurrence of bubbles or other defects, and no deformation or rupturing of the silicone impression occurred.

Some studies use root mean square(RMS) method to evaluate marginal and internal fit [12, 13, 38]. However, these studies may not provide with measurement values of AMD, which is the most clinically meaningful indicators [5, 17]. This study evaluates AMD through specific functions of open source software, aiming to develop corresponding software.

The reliability of the digital evaluation method for the adaptation of restorations has been verified [38]. Li et al. compared measurement results of the digital measurement approach with those of the replica method and reported no statistical difference between the two methods, thereby verifying the reliability of the digital measurement approach in evaluating the fit of restorations [38]. Liang et al. applied the digital measurement approach to evaluate the AMD of single crown, and preliminarily verified its feasibility [17]. It should be noted that, in this experiment, a light-bodied silicone polyether impression material was used to evaluate the fit of FDPs seated on abutments, to seat the restoration without fretting, and to simulate the existence of fluid resistance caused by dental cement in a clinical setting.

A limitation of the present study was that the measurement process involved many interactive operations and required personnel with experience in software operation. For most dentists, there is a notable learning curve. Second, three-unit all-ceramic FDPs were fabricated and measured under in vitro conditions, which cannot fully simulate the effects of oral environment on scanning and impression-making. In addition, this study was limited by the scanning accuracy of the chairside scanner. In the future, various functional modules should be integrated into an automated software platform, thereby simplifying and improving the measurement process, allowing the development of an automated evaluation approach. Such a approach will be beneficial in controlling the quality of marginal fit of FDPs.

## Conclusions

The digital measurement method for absolute marginal discrepancy was preliminarily established based on open source software and applied in three-unit ceramic fixed dental prostheses. The absolute marginal discrepancy of three-unit ceramic fixed dental prostheses fabricated using digital technology was comparable to that of conventional technique.

## Data Availability

All data analyzed and materials used in this study are included in this published article.

## Abbreviations

FDPs: fixed dental prostheses
AMD: absolute marginal discrepancy
STL: standard tessellation language
